# Information and Communication Technologies Interest, Access, and Use: Cross-Sectional Survey of a Community Sample of Urban, Predominantly Black Women

**DOI:** 10.2196/jmir.9962

**Published:** 2018-08-14

**Authors:** Sarah M Jabour, Alexis Page, Seventy F Hall, Lycinda Rodriguez, Wendy C Shields, Anika AH Alvanzo

**Affiliations:** ^1^ Division of General Internal Medicine School of Medicine Johns Hopkins University Baltimore, MD United States; ^2^ School of Social Work University of Buffalo Buffalo, NY United States; ^3^ Department of Psychological, Health and Learning Sciences University of Houston Houston, TX United States; ^4^ Department of Health Policy and Management Bloomberg School of Public Health Johns Hopkins University Baltimore, MD United States

**Keywords:** women, internet communication technology, urban, alcohol, stress, smartphones

## Abstract

**Background:**

Information and communication technologies (ICT) offer the potential for delivering health care interventions to low socioeconomic populations who often face barriers in accessing health care. However, most studies on ICT for health education and interventions have been conducted in clinical settings.

**Objective:**

The aim of this study was to examine access to and use of mobile phones and computers, as well as interest in, using ICT for receipt of behavioral health information among a community sample of urban, predominately black, women with low socioeconomic status.

**Methods:**

Participants (N=220) were recruited from hair salons and social service centers and completed audio-computer assisted self-interviews.

**Results:**

The majority of the participants (212/220, 96.3%) reported use of a cell phone at least weekly, of which 89.1% (189/212) used smartphones and 62.3% (137/220) reported computer use at least weekly. Of the women included in the study, 51.9% (107/206) reported using a cell phone and 39.4% (74/188) reported using a computer to access health and/or safety information at least weekly. Approximately half of the women expressed an interest in receiving information about stress management (51%-56%) or alcohol and health (45%-46%) via ICT. Smartphone ownership was associated with younger age (odds ratio [OR] 0.92, 95% CI 0.87-0.97) and employment (OR 5.12, 95% CI 1.05-24.95). Accessing health and safety information weekly by phone was associated with younger age (OR 0.96, 95% CI 0.94-0.99) and inversely associated with higher income (OR 0.42, 95% CI 0.20-0.92).

**Conclusions:**

Our findings suggest that ICT use, particularly smartphone use, is pervasive among predominantly black women with low socioeconomic status in urban, nonclinical settings. These results show that ICT is a promising modality for delivering health information to this population. Further exploration of the acceptability, feasibility, and effectiveness of using ICT to disseminate behavioral health education and intervention is warranted.

## Introduction

Information and communication technologies (ICT) use has become widespread across the United States. An estimated 73% of American adults own computers and as many as 92% own a cell phone, 68% of which are smartphones, cellular devices with internet capabilities [1]. Although earlier studies found racial and ethnic disparities in ICT accessibility [2-4], recent studies have suggested that this divide is narrowing, particularly related to cell phone use. Black Americans are equally as likely as white Americans to own a cell phone, more likely to report cell phones as a primary internet source and use a wider array of cell phone data functions compared to their white American counterparts [5]. The availability of ICT offers a promising vehicle for dissemination of health education and interventions in a convenient delivery format [6,7].

ICT may be particularly useful for intervening on potentially stigmatizing conditions, such as behavioral health disorders involving substance misuse and/or mental health. Multiple studies have shown that people are more likely to disclose sensitive behaviors when the data are collected using computers as compared to more traditional methods, such as a face-to-face interview [8-10]. Evidence suggests that internet-based interventions can effectively reduce drinking [7,11-13] and are a promising way to improve mental health symptoms [14,15]. This may be especially relevant for minority populations living in low-income, urban neighborhoods, as members of minority populations are less likely to access health care in traditional medical settings; and services for co-occurring mental health and substance misuse are less likely to be found in urban neighborhoods [16-19]. Thus, ICT-based interventions warrant exploration of their potential for reducing health disparities by engaging vulnerable populations in urban medically underserved environments who are often underrepresented in traditional clinical settings [20,21].

Although age, income, and educational attainment remain barriers to accessing health information through ICT [5,21-23], findings suggest that the majority of low-income patients recruited from urban health care settings have access to, and are generally interested in, the use of ICT for health advice and communication with their families’ health providers [22,24]. However, less is known about ICT use and access of behavioral health information among nonclinical urban populations. This exploratory analysis aimed to address gaps in the literature by examining the availability and use of ICT as well as the interest in ICT-based information on alcohol use and stress symptoms management among women recruited from urban community-based sites.

## Methods

### Participants

Participants (N=220) were recruited for a larger study examining the prevalence and relationship between trauma, posttraumatic stress, and high-risk drinking in predominantly black women. Between June 2014 and September 2016, women were recruited from 3 urban hair salons (n=57) and 4 Community Action Partnership Centers (CAPCs). CAPCs are city social service centers providing a variety of resources, including housing and energy assistance (n=163). English speaking women between the ages of 21 to 65 years old were eligible. Women who were pregnant, currently enrolled in treatment for substance use, or unable to provide informed consent were excluded.

### Procedures and Measures

Research assistants approached women in the waiting rooms of the hairs salons and CAPCs. After completing a screening questionnaire, eligible women provided informed consent and used a tablet computer equipped with headphones to complete a battery of instruments via audio-computer assisted self-interview (ACASI). The survey included demographic questions and the Technology Use Survey, a 20-item questionnaire on cell phone, computer, and internet use adapted from an emergency department study examining parents’ access of child health and safety information [24]. The survey was modified to include questions on access to and interest in receiving alcohol and stress management health information via ICT. The study was approved by the Johns Hopkins Institutional Review Board.

### Data Analyses

Descriptive statistics (frequencies, means, and standard errors) were used to describe the sample. Exploratory logistic regression analyses were used to examine the women’s use of ICT based on demographic variables of interest (age, education, employment, and income). The frequency of cell phone and computer use was dichotomized into daily and less than daily use, and frequency of accessing health and safety information was dichotomized into weekly and less than weekly access. Most of the independent variables were binary except age, which was continuous, and education (< high school, high school or general equivalency degree, > high school). Annual household income was also dichotomized (<US $25,000 and ≥US $25,000). Given that this study targeted predominantly black women, race was dichotomized into black and nonblack. However, race was excluded from the logistic regression analyses because it failed criteria for inclusion (*P*<.10) during univariable analyses. Significance was determined at alpha level of .05. Analyses were performed using SPSS version 24.0 for Windows.

## Results

### Demographics

Most women were in their mid-forties (mean [SD] of 44.7 [12.11] years), black (185/220, 84.1%), had at least a high school education (173/220, 78.6%), and reported annual household income of less than US $25,000 (140/220, 64.5%). However, the average household income was largely influenced by women recruited from the CAPCs, as more than half of these women (84/163, 51.5%) reported household incomes of less than US $10,000 annually. When compared to women recruited from the CAPCs, women recruited from salons were roughly four years younger (41.8 vs 45.7; *t=* 2.07, *P*=.04), more likely to be black (*P*=.03), married (χ^2^_2_=15.06; *P*=.001), and employed (χ^2^_1_=33.00; *P*<.001), were more educated (χ^2^_2_=37.47; *P*<.001), and had higher annual household incomes (χ^2^_1_=109.17; *P*<.001). Sample demographic characteristics are presented in Table 1.

### Cell Phone Use

The results from the cell phone section of the questionnaire are presented in Table 2. The overwhelming majority of women (212/220, 96.4%) reported using a cell phone at least weekly. Of those reporting at least weekly cell phone use, the majority reported personal ownership of the phone (206/212, 97.2%), of which a majority were smartphones (189/212, 89.1%). Most women (181/206, 87.9%) had a monthly cell phone plan and reported <5 days in which their phones were not working in the previous three months (180/205, 87.9%). A substantial majority used their cell phones to send or receive emails (145/206, 70.4%) or to access the internet daily (148/206, 71.8%). As shown in Table 2, there were some statistically significant differences for certain characteristics of cell phone use between women recruited from the salons and those recruited from the CAPCs, but not for smartphone ownership or daily internet use via cell phones.

### Computer Use

Two-thirds of the women (147/220, 66.7%) reported at least weekly use of a computer and it was found that the women were accessing computers in multiple locations (Table 3). The home was the most frequent place where participants reported accessing the internet via computer (115/187, 61.5%), followed by work (58/187, 31.0%), and the library (54/187, 28.9%). Just over half reported using the computer to email (97/188, 51.6%) or access the internet (107/188, 56.9%) on a daily basis. Women recruited from salons were more likely to report daily email messaging (41/54, 75.9% vs 56/134, 41.8%; χ^2^_4_=24.48; *P*<.001) and internet access via a computer (42/54, 77.8% vs 65/134, 48.5%; χ^2^_4_=18.59; *P*<.001), respectively, when compared to women recruited from the CAPCs.

**Table 1:** Demographic characteristics of participants.

Characteristic			Total recruited (n=220)		Recruited from salons (n=57)		Recruited from CAPCs^a^ (n=163)		*P* value
Age, mean (SD)			44.7 (12.11)		41.8 (13.0)		45.7 (11.7)		.04
**Race, n (%)**									.03
	Black	185 (84.1)		52 (91.2)		133 (81.6)			
	White	22 (10.0)		1 (1.8)		21 (12.9)			
	Other	13 (5.9)		4 (7.0)		9 (5.5)			
**Education level, n (%)**									<.001
	≤ High school or GED^b^	47 (21.4)		2 (3.5)		45 (27.6)			
	High school or GED	81 (36.8)		12 (21.1)		69 (42.3)			
	>High school	92 (41.8)		43 (75.4)		49 (30.1)			
**Marital status, n (%)**									.001
	Single (never married)	114 (51.8)		25 (43.9)		89 (54.6)			
	Married or living as married	43 (19.6)		21 (36.8)		22 (13.5)			
	Previously married (divorced, separated, or widowed)	63 (28.6)		11 (19.3)		52 (31.9)			
**Employment status, n (%)^c^**									
	Employed for wages	87 (39.9)		41 (71.9)		46 (28.6)		<.001	
	Self-employed	8 (3.7)		2 (3.5)		6 (3.7)		1.00	
	Unemployed	38 (17.4)		5 (8.8)		33 (20.5)		.07	
	Disabled	50 (22.9)		0 (0.0)		50 (31.1)		<.001	
	Homemaker	13 (6.0)		0 (0.0)		13 (8.1)		.02	
	Student	10 (4.6)		4 (7.0)		6 (3.7)		.29	
	Retired	10 (4.6)		5 (8.8)		5 (3.1)		.13	
**Household income, n (%)**									<.001
	<US $25,000	140 (64.5)		3 (5.6)		137 (84.0)			
	≥US $25,000	77 (35.5)		51 (94.4)		26 (16.0)			

^a^CAPC: Community Action Partnership Center.

^b^GED: General Equivalency Diploma.

^c^Respondents able to select multiple options.

**Table 2:** Cell phone use characteristics of participants.

Cell phone use characteristics			Total (n=220)	Salons (n=57)	CAPCs^a^ (n=163)	*P* value
**Do you use a cell phone at least once per week?**						0.12
	No		8 (3.6)	0 (0.0)	8 (4.9)	
	Yes		212 (96.4)	57 (100)	155 (95.1)	
**What kind of cell phone do you use most of the time?^b^**						.33
	Smartphone		189 (89.2)	53 (93.0)	136 (87.7)	
	Other, nonsmartphone		23 (10.8)	4 (7.0)	19 (12.3)	
**Does this phone belong to you or to someone else?^b^**						.20
	Belongs to me		206 (97.2)	57 (100)	149 (96.1)	
	Belongs to someone else		6 (2.8)	0 (0.0)	6 (3.9)	
**How long have you had the same phone number?^c^**						<.001
	≤1 year		57 (27.7)	5 (8.8)	52 (34.9)	
	>1 year		146 (70.9)	49 (86.0)	97 (65.1)	
	Don’t know		3 (1.5)	3 (5.3)	0 (0.0)	
**In the last 3 months, how many days was your phone not working for any reason (disconnected, dead battery, etc)?^c^**						<.001
	0 days		127 (62.0)	47 (82.5)	80 (54.1)	
	1-4 days		53 (25.9)	9 (15.8)	44 (29.7)	
	>5 days		25 (12.2)	1 (1.8)	24 (16.2)	
**What type of cell phone plan do you have?^c^**						.004
	Pay per month		181 (87.9)	56 (98.2)	125 (83.9)	
	Pay as you go (you have to add minutes)		6 (2.9)	1 (1.8)	5 (3.4)	
	Other		19 (9.2)	0 (0.0)	19 (12.8)	
**Thinking just about cell phones, how often do you use a cell phone to:^c^**						
	**Send or get text messages**					.30
		Daily	175 (85.0)	52 (91.2)	123 (82.6)	
		Weekly to monthly	14 (6.8)	3 (5.3)	11 (7.4)	
		Rarely to never	17 (8.3)	2 (3.5)	15 (10.1)	
	**Send or get email messages**					.005
		Daily	145 (70.4)	49 (86.0)	96 (64.4)	
		Weekly to monthly	10 (4.9)	2 (3.5)	8 (5.4)	
		Rarely or never	51 (24.8)	6 (10.5)	45 (30.2)	
	**Access the internet**					.82
		Daily	148 (71.8)	43 (75.4)	105 (70.5)	
		Weekly to monthly	12 (5.8)	3 (5.3)	9 (6.0)	
		Rarely or never	46 (22.3)	11 (19.3)	35 (23.5)	

^a^CAPC: Community Action Partnership Center.

^b^Includes responses only from those reporting at least weekly cell phone use.

^c^Includes responses only from those reporting at least weekly cell phone use and cell phone belongs to them.

**Table 3:** Computer use characteristics of participants.

Computer use characteristics			Total (n=220)	Salons (n=57)	CAPCs^a^ (n=163)	*P* value
**How often do you use a computer for any reason?**						<.001
	Daily		98 (44.5)	44 (77.2)	54 (33.1)	
	Weekly to monthly		49 (22.3)	9 (15.8)	40 (24.5)	
	Rarely to never		73 (33.2)	4 (7.0)	69 (42.3)	
**Where do you use a computer to access the internet?^b,c^**						
	I don't use the internet		10 (5.3)	1 (1.9)	9 (6.7)	.29
	Home		115 (61.5)	40 (75.5)	75 (56.0)	.02
	Work		58 (31.0)	33 (62.3)	25 (18.7)	<.001
	Library		54 (28.9)	6 (11.3)	48 (35.8)	.001
	Other		38 (20.3)	11 (20.8)	27 (20.1)	.40
**Thinking just about computers, how often do you use a computer to:^c^**						
	**Send or get email messages**					<.001
		Daily	97 (51.6)	41 (75.9)	56 (41.8)	
		Weekly to monthly	30 (16.0)	9 (16.7)	21 (15.7)	
		Rarely to never	61 (32.4)	4 (7.4)	57 (42.5)	
	**Access the internet**					<.001
		Daily	107 (56.9)	42 (77.8)	65 (48.5)	
		Weekly to monthly	34 (18.1)	9 (16.7)	25 (18.7)	
		Rarely to never	47 (25.0)	3 (5.6)	44 (32.8)	

^a^CAPC: Community Action Partnership Center.

^b^Respondents able to select multiple options.

^c^Excludes those reporting never using a computer.

### ICT and Access of Health and Safety Information

Approximately half of women reported using a cell phone (107/206, 52.0%) and one-third used a computer (74/188, 39.4%) to access health information at least weekly (Table 4). Just over half were interested in accessing stress management information on their cell phones (115/212, 54.2%) and computers (96/188, 51.5%), and just under half reported interest in accessing alcohol-related health information via cell phones (94/211, 44.5%) and computers (84/188, 44.7%).

### Demographics and Information and Communication Technology Use

The exploratory logistic regression results are shown in Tables 5 and 6. Younger age was associated with all cell phone use and access variables (ie, smartphone ownership, daily text messaging, daily email, daily internet access, and weekly access of health and safety information via cell phone) but not with any of the computer use variables. Compared to women with less than a high school education, women with greater than a high school education were more likely to report daily use of a cell phone to access the internet. Employment was associated with owning a smartphone. Women with annual household incomes of ≥US $25,000 were four times more likely to use a computer daily for email messaging and almost three times more likely to access the internet daily on a computer. There was no association between income and cell phone use with the exception that women with higher incomes were less likely to report weekly access health and safety information via their cell phones.

**Table 4:** ICT (information and communication technology) and access of health and safety information.

Characteristic			Total (n=220)	Salons (n=57)	CAPCs^a^ (n=163)	*P* value
**Thinking just about mobile phones, how often do you use a mobile phone to:**						
	**Get health or safety information^b^**					.10
		Daily	78 (37.9)	15 (26.3)	63 (42.3)	
		Weekly	29 (14.1)	9 (15.8)	20 (13.4)	
		Monthly	20 (9.7)	9 (15.8)	11 (7.4)	
		Rarely to never	79 (38.3)	24 (42.1)	55 (36.9)	
	**Would you like to be able to use a mobile phone for information about dealing with stress?^c^**					.09
		No	97 (45.8)	32 (56.1)	65 (41.9)	
		Yes	115 (54.2)	25 (43.9)	90 (58.1)	
	**Would you like to be able to use a mobile phone to get information about alcohol and health?^c^**						.35
		No	117 (55.5)	35 (61.4)	82 (53.2)	
		Yes	94 (44.5)	22 (38.6)	72 (46.8)	
**Thinking just about computers, how often do you use a computer to:^d^**						
	**Get health or safety information**					.01
		Daily	47 (25.0)	14 (25.9)	33 (24.6)	
		Weekly	27 (14.4)	9 (16.7)	18 (13.4)	
		Monthly	23 (12.2)	13 (24.1)	10 (7.5)	
		Rarely to never	91 (48.4)	18 (33.3)	73 (54.5)	
	**Would you like to be able to use a computer to get information about dealing with stress?**					.75
		No	92 (48.9)	25 (46.3)	67 (50.0)	
		Yes	96 (51.1)	29 (53.7)	67 (50.0)	
	**Would you like to be able to use a computer to get information about alcohol and health?**					.63
		No	104 (55.3)	28 (51.9)	76 (56.7)	
		Yes	84 (44.7)	26 (48.1)	58 (43.3)	

^a^CAPC: Community Action Partnership Center.

^b^Includes responses only from those reporting at least weekly cell phone use and cell phone belongs to them.

^c^Includes responses only from those reporting at least weekly cell phone use.

^d^Excludes those reporting never using a computer.

**Table 5:** Demographic associations with cell phone use.

Characteristic		Smartphone ownership, OR^a^ (95% CI)	Daily text messages, OR (95% CI)	Daily email messages, OR (95% CI)		Daily internet access, OR (95% CI)		Weekly health/safety info, OR (95% CI)	
Age		0.92 (0.87-0.97)^b^	0.88 (0.83-0.93)^b^	0.93 (0.90-0.96)^b^		0.89 (0.85-0.93)^b^		0.96 (0.94-0.99)^b^	
**Education**									
	<High school or GED^c^ (reference)	1.00	1.00		1.00		1.00		1.00
	High school or GED	0.45 (0.13-1.55)	1.06 (0.35-3.25)		1.26 (0.54-2.95)		1.18 (0.47-2.96)		1.30 (0.58-2.94)
	>High school	0.99 (0.21-4.65)	0.52 (0.15-1.85)		2.09 (0.78-5.61)		5.10 (1.62-16.00)^b^		2.42 (0.99-5.93)
**Employment^d^**									
	Not working (reference)	1.00	1.00		1.00		1.00		1.00
	Working	5.12 (1.05-24.95)^b^	2.28 (0.71-7.30)		1.24 (0.55-2.80)		1.23 (0.50-3.05)		1.85 (0.91-3.74)
**Income**									
	<US $25,000 (reference)	1.00	1.00		1.00		1.00		1.00
	≥US $25,000	1.07 (0.27-4.22)	3.34 (0.96-11.59)		2.50 (1.00-6.26)^e^		0.57 (0.20-1.57)		0.42 (0.20-0.92)^b^

^a^OR: odds ratio.

^b^Statistically significant.

^c^GED: General Equivalency Diploma.

^d^Working defined as employed for wages and self-employed; not working defined as out of work, homemaker, student, retired, or disabled.

^e^Appears statistically significant only because of rounding.

**Table 6:** Demographic associations with computer use.

Characteristic		Daily email messages, OR^a^ (95% CI)	Daily internet access, OR (95% CI)	Weekly health or safety info, OR (95% CI)
Age		1.00 (0.97-1.03)	1.00 (0.97-1.02)	1.00 (0.97-1.02)
**Education**				
	<High school or GED^b^ (reference)	1.00	1.00	1.00
	High school or GED	0.98 (0.38-2.52)	0.88 (0.35-2.21)	1.57 (0.58-4.22)
	>High school	1.48 (0.55-3.96)	1.43 (0.54-3.78)	2.38 (0.86-6.57)
**Employment^c^**				
	Not working (reference)	1.00	1.00	1.00
	Working	1.62 (0.79-3.32)	1.85 (0.90-3.78)	1.06 (0.52-2.13)
**Income**				
	<US $25,000 (reference)	1.00	1.00	1.00
	≥US $25,000	4.00 (1.88-8.51)^d^	2.87 (1.34-6.15)^d^	1.26 (0.62-2.59)

^a^OR: odds ratio.

^b^GED: General Equivalency Diploma.

^c^Working defined as employed for wages and self-employed; not working defined as out of work, homemaker, student, retired, or disabled.

^d^Statistically significant.

## Discussion

### Principal Results and Comparison with Prior Work

In this sample of predominantly black women with low socioeconomic status recruited from urban hair salons and social service centers, we found high rates of ICT access and use, particularly with regard to smartphone use. Additionally, we found a moderate use of ICT to access health and safety information and moderate interest in receipt of behavioral health information on alcohol or stress management via ICT. Younger age was associated with smartphone ownership, daily internet access, and weekly access of health and safety information. We found that employment was associated with smartphone ownership, and higher educational attainment was associated with daily internet access via cell phones. Our findings are consistent with other studies demonstrating associations between technology use, age [5,21,25], and educational attainment [5,21,22,25]. Higher income was associated with daily email messaging and internet access via computer and inversely associated with weekly access of health and safety information by cell phone. Notably, smartphone ownership was not associated with income. Our results suggest that ICT health interventions would be accessible and may be of interest as a modality for receipt of behavioral health information in women recruited from urban community sites. These findings support consideration and exploration of the use of ICT, particularly smartphones, as a tool to educate women outside of traditional healthcare delivery settings.

With the evolution of cell technology, portable ICT devices have many of the same functionalities as computers, potentially decreasing the need for a computer. The ICT modality most frequently used by the women in our sample was cell phones, particularly smartphones. While 62.2% of women in our sample reported at least weekly computer use, 96.4% were using a cell phone at least weekly. The near universal report of cell phone ownership and use among our community-based sample is consistent with that of the general US population [1] and clinic-based samples of urban predominantly lower socioeconomic status patients [22,24-28]. Yet with respect to smartphone ownership, our findings differed from previous analyses. A larger proportion of women in our sample reported smartphone use (89%) compared to the most recent general population survey of US adults (68%) [1]. In our sample, cell phones were the principal ICT means for communicating which is consistent with other studies of persons with lower socioeconomic status [22,24,29]. These results are also consistent with national data indicating that racial and ethnic minorities, specifically black and Latino Americans, lead the way with respect to use of cell devices for accessing the internet, social media sites, health information, and tracking or managing health with specialized apps [20,30]. Reasons for these differences are likely multifactorial including decreased rates of home broadband access and tablet ownership for black Americans compared to white Americans, making cell phones the only device available for internet use [31,32], as well as racial differences in attitudes about information exchange via cell devices [33-35].

More than half of the women in the sample used ICT to access health and safety information at least weekly. About half of the participants in this study reported interest in receiving information about stress management and/or alcohol and health via ICT. These rates are somewhat lower than other studies among low socioeconomic samples conducted in clinical settings which focused on the receipt of general medical information rather than behavioral health information. For example, Mitchell and colleagues found that the majority of parents (84%) in pediatric clinics were willing to receive health information through ICT [22]. Studies specifically examining interest in ICT for behavioral health education or intervention have reported variable results. Torous and colleagues found that approximately half of the patients (49%) recruited from 4 psychiatric clinics across the US reported accessing general health information via smartphone in the previous 6 months, but approximately 71% reported interest in using a smartphone to monitor mental health symptoms [36]. In contrast, Sharpe and colleagues found that only 29% of persons living with HIV, who were recruited from 5clinical sites in Florida, reported interest in using a cell phone app to self-manage drinking, though there were significant differences based on respondents’ drinking levels, with those with hazardous drinking being more likely to express interest [37]. Our results highlight that interest in and use of ICT for accessing health-related information extend beyond clinic-based samples and support the acceptability of ICT-based behavioral health educational applications, including those focused on stress management or alcohol use.

### Limitations

These findings should be interpreted in the context of the study’s limitations. The sample size was small and not nationally representative, which limits generalization of our results to men, nonblack racial groups, or nonurban populations. Analyses relied on self-reported data, which introduced potential response bias, as participants may be more likely to provide socially desirable answers, and we did not have procedures to confirm ICT use. This may have been mitigated by our use of ACASI. We did not inquire about use of tablet computers or downloadable apps for cell phones, which limited our ability to report on use or acceptability of these ICT formats for delivering health information and interventions.

Despite these limitations, this study extends the existing literature on ICT use. This study advances the literature by surveying a population that been underrepresented in previous surveys on ICT access or interests. The unique method of sampling from urban hair salons and CAPCs provides valuable new information about ICT use and the potential for health-related education among a community sample of predominantly black women with low socioeconomic status. Additionally, this study used a computer-assisted survey, which is a unique method among studies of populations with low socioeconomic statuses. Our results suggest that smartphone behavioral health education programs would be both accessible and of interest to black women in urban areas with low socioeconomic status.

### Implications for Practice and Policy

There are several potential advantages of ICT-delivered health education and intervention applications. ICT interventions allow for increased intervention fidelity as unlike humans the software’s intervention delivery will be standardized [7,38]. ICTs offer a more cost-effective approach to education and or intervention delivery as compared to employing a full-time health educator or interventionist [39-41]. ICTs allow for increased confidentiality and elimination of barriers to care associated with traditional interventions, such as discomfort discussing sensitive topics [6,38]. Finally, the widespread adoption of ICT use offers increased portability, reach and convenience, allowing patients to complete the education and/or intervention at times most suitable to them, as well as eliminating potential barriers of lack of transportation and lapses in health insurance [6,38,42,43]. Future research should explore ICT app use, preferred design and content features as well as the development, piloting, and assessment of utility and cost-effectiveness of different ICT health-related modalities in this population.

### Conclusions

ICT, particularly smartphones, were widely available and used in our sample of urban, predominantly black women with low socioeconomic status. These devices offer a promising vehicle to study the delivery of behavioral health education and intervention in this population. Given these findings, developers of ICT-based behavioral health programs should ensure cell platforms are as robustly developed and investigated as computer-based modalities.

## Abbreviations

ACASI: audio-computer assisted self-interview
CAPC: Community Action Partnership Center
GED: General Equivalency Diploma
ICT: information and communication technology
OR: odds ratio
